# Alterations in Spontaneous Brain Oscillations during Stroke Recovery

**DOI:** 10.1371/journal.pone.0061146

**Published:** 2013-04-11

**Authors:** Kristina Laaksonen, Liisa Helle, Lauri Parkkonen, Erika Kirveskari, Jyrki P. Mäkelä, Satu Mustanoja, Turgut Tatlisumak, Markku Kaste, Nina Forss

**Affiliations:** 1 Brain Research Unit, O.V. Lounasmaa Laboratory and MEG Core, Aalto Neuroimaging, Aalto University, Aalto, Espoo, Finland; 2 Department of Neurological Sciences, University of Helsinki, HY, Helsinki, Finland; 3 Department of Neurology, Helsinki University Central Hospital, HUS, Helsinki, Finland; 4 Elekta Oy, Helsinki, Finland; 5 Department of Biomedical Engineering and Computational Science, Aalto University School of Science, Aalto, Espoo, Finland; 6 Department of Clinical Neurophysiology, HUS Medical Imaging Center, Helsinki University Central Hospital, HUS, Helsinki, Finland; 7 BioMag Laboratory, HUS Medical Imaging Center, Helsinki University Central Hospital, Helsinki, Finland; McGill University, Canada

## Abstract

Amplitude or frequency alterations of spontaneous brain oscillations may reveal pathological phenomena in the brain or predict recovery from brain lesions, but the temporal evolution and the functional significance of these changes is not well known. We performed follow-up recordings of spontaneous brain oscillations with whole-head MEG in 16 patients with first-ever stroke in the middle cerebral artery territory, affecting upper limb motor function, 1–7 days (T_0_), 1 month (T_1_), and 3 months (T_2_) after stroke, with concomitant clinical examination. Clinical test results improved significantly from T_0_ to T_1_ or T_2_. During recovery (at T_1_ and T_2_), the strength of temporo–parietal ∼10-Hz oscillations in the affected hemisphere (AH) was increased as compared with the unaffected hemisphere. Abnormal low-frequency magnetic activity (ALFMA) at ∼1 Hz in the AH was detected in the perilesional cortex in seven patients at T_0_. In four of these, ALFMA persisted at T_2_. In patients with ALFMA, the lesion size was significantly larger than in the rest of the patients, and worse clinical outcome was observed in patients with persisting ALFMA. Our results indicate that temporo–parietal ∼10-Hz oscillations are enhanced in the AH during recovery from stroke. Moreover, stroke causes ALFMA, which seems to persist in patients with worse clinical outcome.

## Introduction

Magnetoencephalography (MEG) and electroencephalography (EEG) studies have shown that the human brain exhibits spontaneous intrinsic electrical oscillations at various frequencies. In healthy brain, the most prominent oscillations occur in the 8–30 Hz frequency range and their quantitative parameters such as frequency and power have been shown to be intraindividually rather stable across repeated measurements [1], [2]. Although the functional significance of these brain rhythms is not thoroughly understood, changes in the amplitude or frequency as well as alterations in task-related modulation of brain rhythms, have been linked with pathological phenomena of the brain [3]–[6].

Several EEG [4], [6], [7] and MEG studies [5], [8]–[10] have reported changes in spontaneous brain oscillations after stroke. However, the results are highly variable: for example, both attenuation and enhancement of ipsilesional rolandic ∼10-Hz activity has been observed, and the temporal evolution from the acute phase and the association of these findings with clinical recovery are unclear [4], [6], [9]. Moreover, gamma-band power in the affected hemisphere and delta-band power in the unaffected hemisphere in the acute phase after stroke have been suggested to predict clinical outcome [5].

In addition to alterations in physiological brain oscillations, abnormal low-frequency magnetic activity (ALFMA, in the 0.5–6 Hz range) has been reported in patients with brain lesions such as traumatic brain injury [3], [11] and stroke [12], [13]. However, the association of ALFMA with clinical outcome is not yet known.

The aim of this study was to investigate 1) how the earlier reported alterations in rolandic ∼10-Hz oscillations evolve during recovery from stroke and if these alterations are associated with functional recovery, 2) if alterations in spontaneous brain oscillations can predict recovery from stroke and 3) how ALFMA is associated with clinical recovery from stroke. To achieve this, we measured spontaneous resting state brain oscillations with a whole-head MEG in 20 first-ever stroke patients within one week and one and three months after stroke. Compared with EEG, MEG has the advantage that the brain signals are practically unaffected by the conductivity differences of the brain and surrounding tissues, giving MEG a better spatial accuracy. With whole-head MEG, the activity of the two hemispheres can be recorded simultaneously and thus compared in exactly same conditions.

## Materials and Methods

### Patients and control subjects

Twenty patients with a first-ever acute stroke in the middle cerebral artery territory affecting upper limb motor function were recruited at the Department of Neurology, Helsinki University Central Hospital (HUCH), Helsinki, Finland. Exclusion criteria were earlier neurological diseases, head traumas or neurosurgical operations, severe psychiatric disorders, and unstable or poor general condition. Magnetoencephalographic (MEG) recordings were performed in the BioMag Laboratory, HUCH, within 1–7 (mean 3.5±0.5) days (T_0_) and after one (T_1_) and three (T_2_) months from stroke onset. One patient was excluded because of a recurrent infarction after the first measurement, and the MEG data of three patients had to be excluded because of technical problems in the recordings, preventing reliable analysis. Thus the data of 16 patients (8 females, 8 males; age 44–84 years, mean age 66±3 years; all right-handed) and ten healthy control subjects (5 females, 5 males; mean age 61±2 years, all right-handed) were used for further analysis. One of the included patients refused the third measurement (T_2_) because of claustrophobia, whereas the rest of the patients underwent successfully all three measurement sessions.

The study was approved by the Ethics Committees of the Helsinki and Uusimaa Hospital District. All patients and control subjects gave written informed consent. Somatosensory evoked fields (SEFs) to tactile finger stimulation and motor cortex excitability of the same patients and control subjects have been reported previously [14]–[16].

### Clinical and neuroradiological evaluation

Clinical examination was performed at T_0_, T_1_, and T_2_. It consisted of National Institutes of Health Stroke Scale (NIHSS; 0–42 points, with 0 being the best score), modified Rankin Scale (mRS; 0–5 points, with 0 being the best score), and Nine Hole Pegboard test (Peg; time measured to remove and replace nine pegs as fast as possible; upper limit set to 120 s) scoring. To evaluate the size and site of ischemic lesion, anatomical MRIs with T1 MPRAGE and T2 sequences were taken with a 3 T MR scanner (Philips) at T_0_ and T_1_.

### Magnetoencephalographic (MEG) recordings

Spontaneous brain activity during rest was recorded with a 306-channel whole-head MEG device (Elekta Neuromag®, Elekta Oy, Helsinki, Finland), housing 204 gradiometer and 102 magnetometer sensors, in a magnetically shielded room. During the recordings, the subjects were, according to their clinical condition and their own wish, either sitting or lying with the head resting on the sensor helmet. The subjects were asked to relax and not to move their head. During the recordings, a nurse inside the magnetically shielded room observed the patients' general condition. The exact head position with respect to the sensor array was determined at the beginning of each measurement by detecting the magnetic fields produced by four indicator coils placed on the scalp. To align the MEG data with the coordinate system of anatomical MRIs, the locations of the indicator coils with respect to anatomical landmarks were determined with a three-dimensional digitizer before each measurement session. During the 6-min recording, subjects kept their eyes open/closed for 3 min each. The MEG signals were sampled at 941 Hz and band-pass filtered to 0.03–308 Hz.

### Data analysis

To remove interference due to external and nearby artifact sources, the data were first processed with the temporal extension of the signal space separation method (tSSS) [17], [18] implemented in Maxfilter™ software (Elekta Oy) using a correlation window length of 16 s and a correlation limit of 0.98. In each patient, the data from measurements at T_0_ and T_1_ were transformed to correspond to the head position in the measurement at T_2_ allowing a more accurate sensor-level comparison of location and strength of spontaneous brain activity. To remove the cardiac contamination occurring at the frequency band of ALFMA, a signal-space projection [19] was derived by averaging the MEG signals with respect to the magnetocardiographic QRS complex, applying principal component analysis to the average, and selecting the two principal components associated with the highest singular values. These components were projected out from the tSSS-processed continuous data.

**Figure 1:**
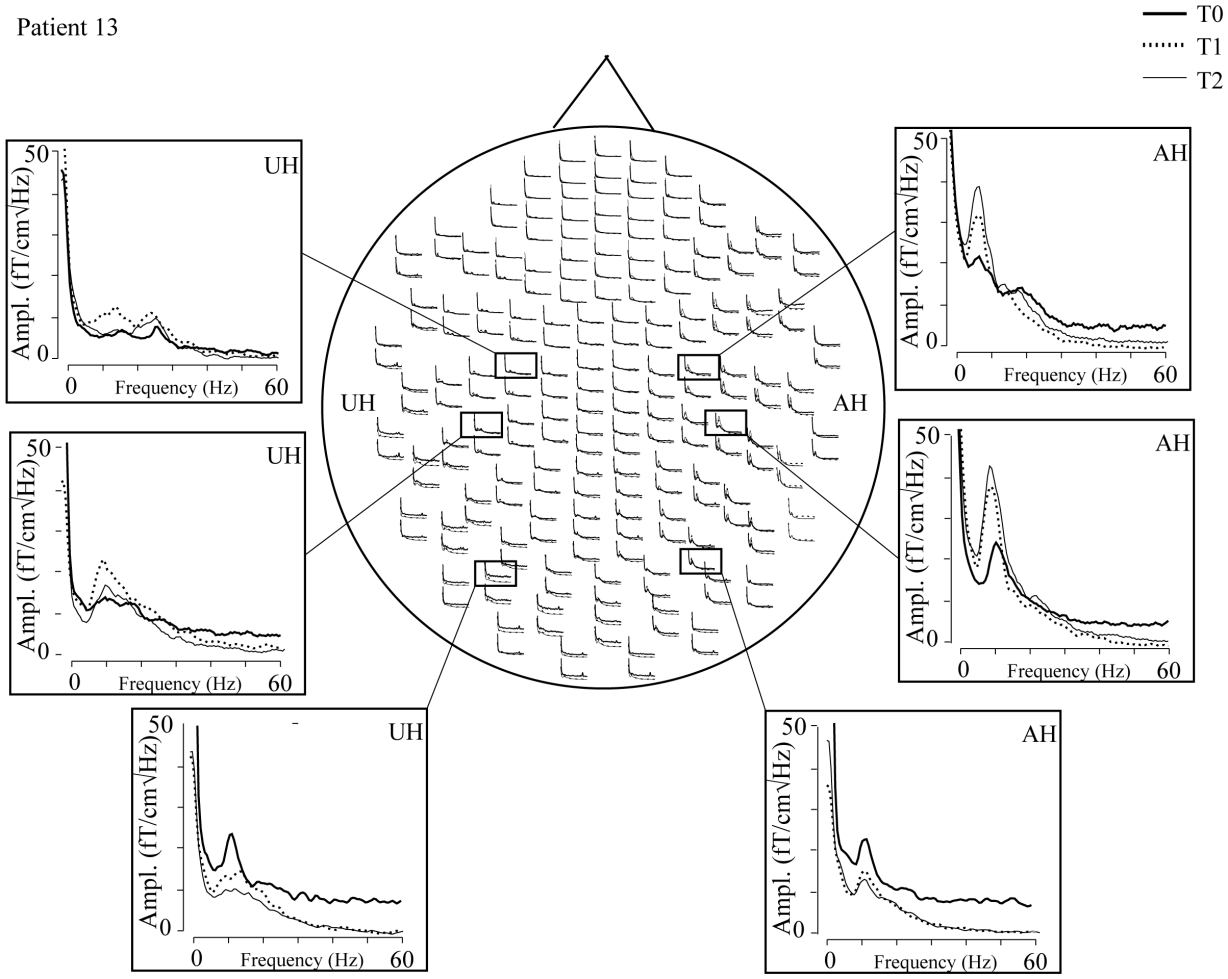
Amplitude spectra of one patient (gradiometers; eyes open). Three channels from both hemispheres, showing peaks of rolandic ∼10-Hz, rolandic beta, and occipital alpha activity. Rolandic ∼10-Hz oscillations are clearly increased in the affected hemisphere (AH). UH; unaffected hemisphere. T_0_, 1 week; T_1_, 1month; T_2_, 3 months after stroke.

After preprocessing the data, the amplitude spectra of spontaneous brain activity were calculated separately for the eyes-open and eyes-closed conditions by averaging the magnitudes of fast Fourier transforms (FFT) in half-overlapping windows over the whole measurement time. To estimate the amplitudes of spontaneous brain oscillations in the frequency range of 5–90 Hz, 2048-point FFTs, corresponding to a frequency resolution of ∼0.5 Hz were used. A flat-top window was applied in conjunction with the FFTs to get accurate amplitude estimates from the spectra. To identify possible ALFMA (0–4 Hz), 8192-point FFTs, resulting in a frequency-resolution of ∼0.1 Hz, with a Hanning window were used. A Hanning window was chosen for its better frequency resolution compared to the flat-top window. The amplitudes of the spectral peaks over the centroparietal (rolandic 10-Hz and beta rhythms) and parieto–occipital (alpha rhythm) regions were quantified from 5–9 MEG channels showing the largest amplitudes (see Fig. 1).

Minimum current estimates in the frequency domain (fdMCE) [20] were calculated to localize the sources of spontaneous brain oscillations. fdMCE for ∼10-Hz activity was calculated for the eyes-open condition to avoid the contaminating effect of strong occipital alpha activity. In fdMCE, 2048-point Hanning windowed half-overlapping FFTs (frequency resolution of ∼0.5 Hz) were computed across the recordings, and the frequency components in individual frequency windows (detected in the individual spectra) were source-modeled using L1 minimum norm estimation. A realistically-shaped standard brain volume with a uniform 10-mm grid was used as the source space, and a spherical conductor model was used for the forward computations. The source locations were projected to the surface of the standard brain volume for visualization, and the strength of the ∼10-Hz activity at each subjects' individual peak frequency was defined for each hemisphere in a region of interest (ROI) which excluded the parieto–occipital region. The same ROI was used for each subject and each measurement, and symmetrical (with respect to the interhemispheric plane) ROIs were used for the left and right hemispheres. The interhemispheric ratio of ∼10-Hz oscillations was calculated with the formula (AH/UH)*100 and compared between different time points and between patients and control subjects. The source locations of ALFMA were estimated from the eyes-closed condition to minimize possible artifacts due to eye blinks, which contaminate the same frequency band. The procedure was as above, except that 4096-point Hanning-windowed FFTs were computed to achieve a frequency resolution of ∼0.2 Hz. For each patient, an individual ROI was defined to enclose the detected activity in all three measurements, and the same ROI was used within one patient to evaluate the persistence of ALFMA. The strength of ALFMA in the affected hemisphere was normalized by subtracting the strength in the mirrored ROI in the unaffected hemisphere. For comparison, the sources of ALFMA were modeled in two patients also with equivalent current dipoles (ECDs) fitted sequentially in 10-ms steps to band-pass-filtered (0–3 Hz) data using a subset of ∼40 channels over the area with largest signals. Only ECDs that explained at least 92% of the measured field pattern were accepted.

Repeated-measures ANOVA with within-subject factors time (T_0_, T_1_, T_2_) and hemisphere (affected, AH; unaffected, UH), and for the beta-range a three-way ANOVA with the additional within-subject factor beta-range (beta_1_, beta_2_), were used to evaluate alterations of frequencies and amplitudes of spontaneous brain oscillations, and to analyze the clinical tests. When a significant main effect was detected, pair-wise comparisons were performed between different time points or between hemispheres. Bonferroni-corrected independent sample t-tests were used to compare the parameters between patients and control subjects. Clinical parameters between patients with *vs*. without ALFMA were compared with non-paired t-tests. A *p*-value <0.05 was considered statistically significant.

## Results

### Clinical outcome

Patients' clinical details are summarized in Table 1. The patients recovered well: all measured clinical parameters improved significantly from T_0_ to T_1_ or T_2_ (Peg: 81±10 s *vs*. 57±10 s and 48±8 s; NIHSS: 4±1 *vs*. 2±1 and 1±0; mRS: 3±0 *vs*. 2±0 and 2±0, *p*<0.01 for all). The improvement was steepest from T_0_ to T_1_. Although the overall clinical outcome, measured with NIHSS and mRS, improved still from T_1_ to T_2_ (*p*<0.05), no significant improvement of hand dexterity, as measured with Peg, was observed from T_1_ to T_2_. However, the affected hand function did not reach the level of the healthy hand function during our three-month follow-up (48±8 s *vs*. 26±1 s, *p*<0.05).

**Table 1:** Clinical details of the patients.

Pat.	1	2	3	4	5	6	7	8	9	10	11	12	13	14	15	16
Gender	M	F	M	M	M	M	M	F	M	F	F	F	F	F	F	M
Age	67	55	78	57	68	62	60	72	74	84	67	68	74	72	48	61
AH	R	R	L	R	L	L	R	L	L	R	L	L	R	L	L	R
Site	CS	CS	S	CS	CS	CS	C	C	C	C	S	S	S	S	S	S
Size	7	70	10	106	48	5	0.1	0.3	0.4	1	1	3	5	3	1	4
Peg, T_0_	120	120	120	120	58	47	120	32	41	120	38	120	120	36	33	56
Peg, T_2_	120	120	32	81	31	33	43	24		44	27	24	39	29	21	51
NIHSS, T_0_	9	9	3	4	3	4	2	3	1	1	2	4	7	1	2	5
NIHSS, T_2_	1	6	1	3	2	1	0	0		0	2	0	1	1	1	2
mRS, T_0_	4	5	5	5	2	5	3	1	2	2	2	5	5	3	2	2
mRS, T_2_	3	3	1	2	2	2	1	1		1	1	2	3	1	2	2

AH, affected hemisphere. C, cortical. CS, cortico-subcortical. S, subcortical. Size, lesion volume in cm^3^. St, Peg, time (s). NIHSS; National Institutes of Health Stroke Scale (0–42 points). mRS, modified Rankin Scale (0–5 points). T_0_, 1–7 days; T_2_, 3 months after stroke.

### Spontaneous brain rhythms

#### Temporo–parietal area

Figure 1 shows the spectra of spontaneous brain activity in gradiometers (0–60 Hz, eyes open) in one patient at T_0_, T_1,_ and T_2_. Spectral peaks are observed at ∼10 Hz and at ∼20 Hz over the temporo–parietal region in both hemispheres and at ∼10 Hz over the occipital region. In all patients and control subjects, 2 to 3 spectral peaks were observed over the temporo–parietal region in both hemispheres; around 9 Hz (corresponding to rolandic 10-Hz rhythm), around 15 Hz (beta_1_), and around 20 Hz (beta_2_; Table 2). However, distinct beta_1_ and beta_2_ peaks were not found in all patients and control subjects. No systematic spectral peaks at frequencies higher than ∼20 Hz were observed in the control subjects or in the patients. No significant differences in any of the frequencies were found between the hemispheres of the control subjects. Neither were there differences in the peak frequencies over the temporo–parietal region between the hemispheres nor measurements of the patients, nor between patients and control subjects.

**Table 2:** Frequencies (Hz) and amplitudes of spectral peaks in the patients (fT/cm√Hz) over the temporo-parietal (eyes open) and occipital (eyes closed) areas.

	temporo-parietal						occipital	
	∼10 Hz		beta 1		beta 2		∼10 Hz	
	AH	UH	AH	UH	AH	UH	AH	UH
f, T_0_	9.2±0.4	9.2±0.3	14.8±0.4	15.4±0.4	20.1±0.4	20.6±0.4	8.4±0.2	8.7±0.2
f, T_1_	9.1±0.5	9.0±0.3	14.9±0.5	15.0±0.5	20.4±0.5	22.3±0.7	8.7±0.2	8.9±0.2
f,T_2_	9.0±0.4	9.2±0.4	15.1±0.3	15.3±0.3	20.8±0.5	20.6±0.4	**8.6±0.2**	**9.1±0.2**
f, Ctrl		9.2±0.2		14.6±0.2		18.4±0.3		8.9±0.2
A,T_0_	34±7	30±4	26±3	28±3	19±2	22±2	46±9	48±9
A,T_1_	**35±3**	**27±2**	30±4	28±3	24±3	22±2	53±6	53±7
A,T_2_	**35±3**	**26±2**	29±3	25±3	25±3	23±2	52±5	43±6
A,Ctrl		32±3		26±2		22±2		57±8

AH, affected hemisphere. UH, unaffected hemisphere. f, frequency (Hz). A, amplitude (fT/cm√Hz) T_0_, 1–7 days; T_1_, 1 month, and T_2_, 3 months after stroke. Ctrl., control subjects (right and left hemispheres pooled).

In the patients, at sensor level, the amplitude of the major spectral peak at ∼10 Hz over the temporo–parietal region (eyes open) was significantly stronger in the AH than in the UH at T_1_ and T_2_. Repeated-measures ANOVA showed a significant main effect for the factor hemisphere [F(1,15) = 5.721, *p*<0.05]. Pair-wise comparison showed that the amplitude of the ∼10-Hz rhythm was significantly stronger in the AH than in the UH at T_1_ and at T_2_ (*p*<0.05 and *p*<0.005, respectively; Table 2).

**Figure 2:**
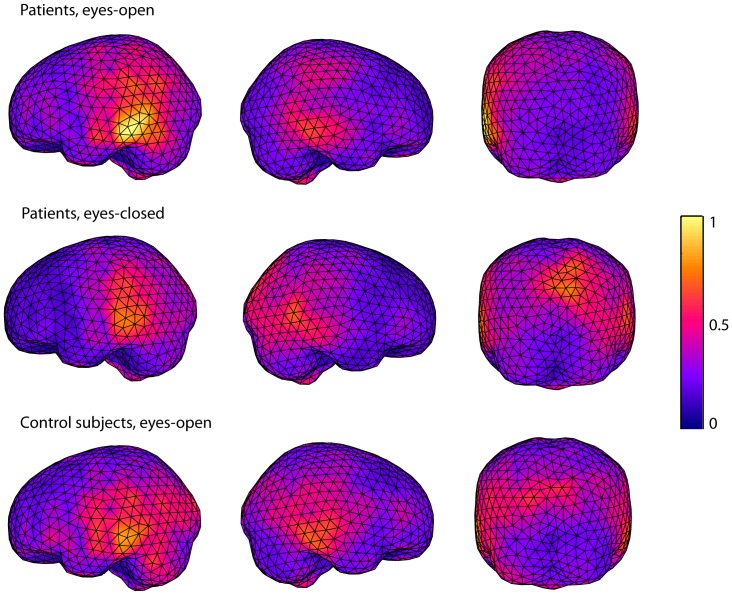
Source localizations of ∼10-Hz oscillations in patients with left hemispheric stroke and in control subjects. Averages of source localizations of ∼10-Hz oscillations (arbitrary scale) estimated with fdMCE in the patients with left hemispheric stroke (eyes-open/eyes-closed) and in the control subjects (eyes-open).

**Figure 3:**
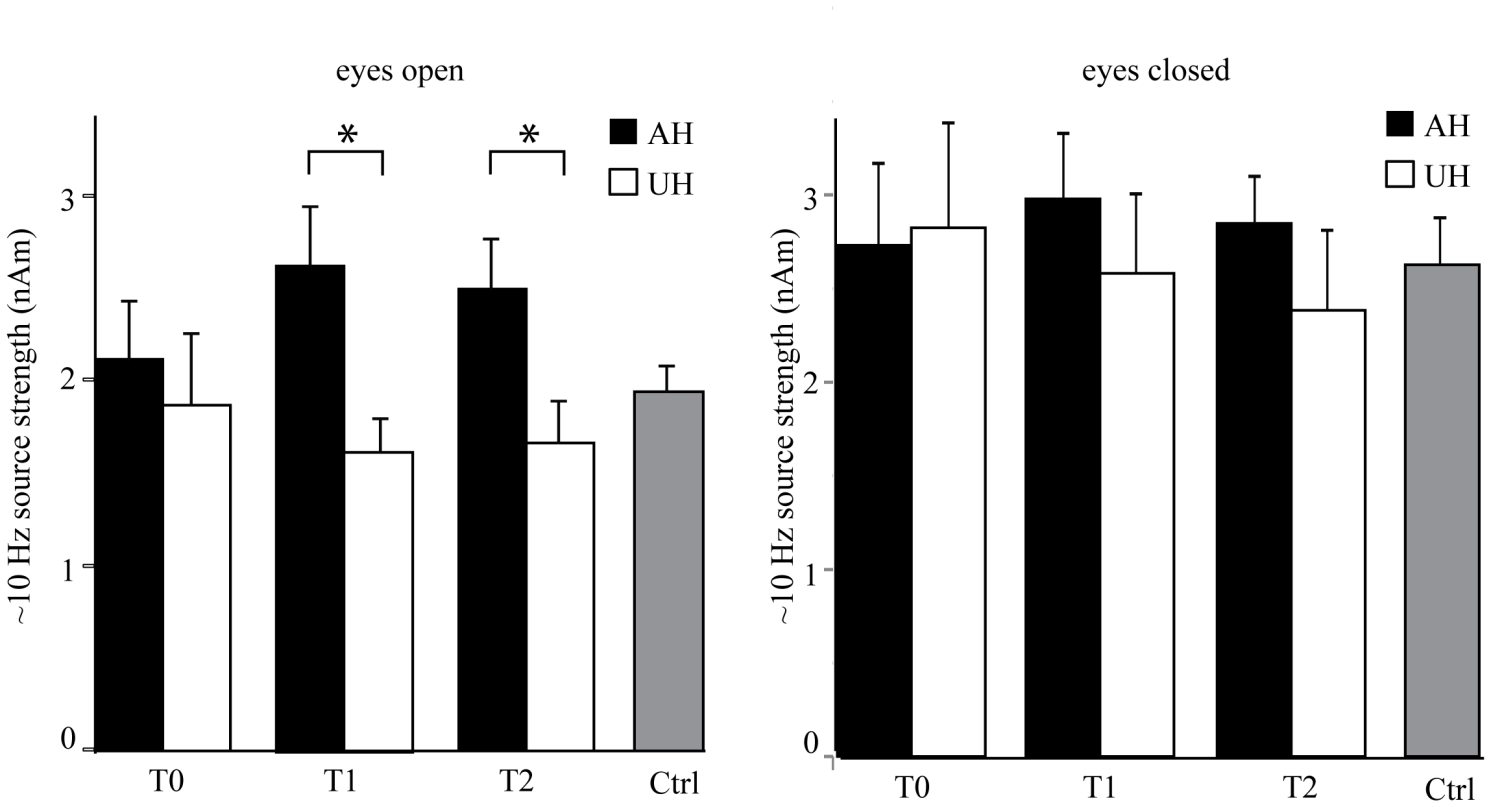
Mean (+SEM) source strength of ∼10-Hz oscillations over the temporo–parietal region (eyes open/eyes closed). AH, affected hemisphere. UH, unaffected hemisphere. T_0_, 1 week; T_1_, 1month; T_2_, 3 months after stroke. *p<0.05.

**Figure 4:**
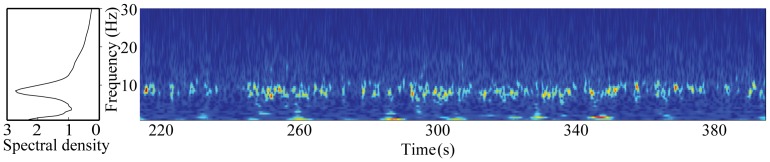
Spectrum and time-frequency representation of ∼10-Hz oscillations from one channel over the temporo–parietal region in one patient (eyes open). The amplitude spectrum of the channel is shown on the left (spectral density in arbitrary units). The burst-like nature of ∼10-Hz oscillations is illustrated in the time-frequency representation on the right.

Figure 2 shows the averages of the source locations of ∼10-Hz oscillations in the patients with a left hemispheric stroke and in the control subjects. The strongest sources for the eyes-open condition are in the temporo–parietal region, clearly distinct from occipital alpha sources, but slightly lateral to the typical location of rolandic ∼10-Hz oscillations. In the patients' eyes-open condition, the sources of ∼10-Hz oscillations are clearly stronger in the AH than in the UH. Such difference is not seen in the eyes-closed condition, or in the control subjects. Repeated-measures ANOVA showed a significant main effect for the factor hemisphere [F(1,12) = 5.590, *p*<0.05] and an interaction between the factors time and hemisphere [F(2,24) = 3.556, *p*<0.05] for the source strength. Pair-wise comparison showed that the mean strength of the sources of ∼10-Hz oscillations was significantly stronger in the AH than in the UH at T_1_ and at T_2_ (*p*<0.05, Figure 3, Table 3). The amplitude of the ∼10-Hz sources seemed to increase in bursts (Figure 4), varying strongly in source localization and strength from time window to another, used in the spectral estimation. As fdMCE calculates the mean strength of all the FFT-windows, the absolute values for the ∼10-Hz sources remain rather small.

**Table 3:** Source strength estimates (nAm) of ∼10-Hz activity over the temporo-parietal region in all patients (mean ± SEM), and low-frequency (∼1-Hz) oscillations in the patients, in whom low-frequency oscillations were detected (mean ± SEM).

	∼10 Hz, AH		∼10 Hz, UH		∼ 1 Hz, AH
	eyes–open	eyes–closed	eyes–open	eyes–closed	eyes–closed
T_0_	2.1±0.4	2.7±0.4	1.9±0.4	2.8±0.6	**8±2**
T_1_	**2.6±0.3**	**3.0±0.4**	1.6±0.2	2.6±0.4	**6±1**
T_2_	**2.5±0.3**	**2.8±0.3**	1.7±0. 2	2.4±0.4	**4±1**
Ctrl			1.9±0.2	2.6±0.3	

AH, affected hemisphere. UH, unaffected hemisphere. T_0_, 1–7 days; T_1_, 1 month, and T_2_, 3 months after stroke. Ctrl., control subjects (hemispheres pooled).

ANOVA showed a main effect for the interhemispheric ratio (AH/UH) of the strength of ∼10-Hz oscillations [F(2,24) = 3.865, *p*<0.05]. Pair-wise comparison showed that the interhemispheric ratio was larger at T_1_ and T_2_ than at T_0_ (178% and 180% *vs*. 129%, *p*<0.05, respectively). No significant differences in the strength of the temporo–parietal ∼10-Hz rhythm was detected between the hemispheres of the control subjects or between patients and control subjects. However, the interhemispheric ratio (AH/UH) of the strength of ∼10-Hz oscillations was significantly larger in the patients at T_1_ and T_2_ than in the control subjects (178% and 180% *vs*. 93%, *p*<0.05, respectively). The source strength of the ∼10-Hz rhythm for the eyes-closed condition did not differ between the hemispheres, or between the measurements. Peak amplitudes of the beta_1_ (∼15 Hz) and beta_2_ (∼20 Hz) rhythms, generated in the rolandic region, did not differ between the hemispheres, time points, or patients and control subjects (Table 2).

In summary, both amplitude spectra and fdMCE revealed that the strength of ∼10-Hz oscillations in the AH was enhanced at T_1_ and T_2_ as compared with the UH.

#### Occipital area

The spectral peaks over the occipital region (eyes closed, corresponding to occipital alpha) are listed in Table 2. For the peak frequency of the occipital alpha, ANOVA showed a significant main effect for the factor hemisphere [F(1,12) = 21.309, *p*<0.001]. Pair-wise comparison showed that the peak frequency of the occipital alpha was significantly lower in the AH than in the UH at T_2_ (*p*<0.05). At T_0_ and T_1_, the difference was not significant (*p* = 0.12 and *p* = 0.11, respectively). The amplitudes of the occipital ∼10-Hz rhythm did not differ between the hemispheres, measurements, or patients and control subjects.

### Abnormal low-frequency magnetic activity (ALFMA)

**Figure 5:**
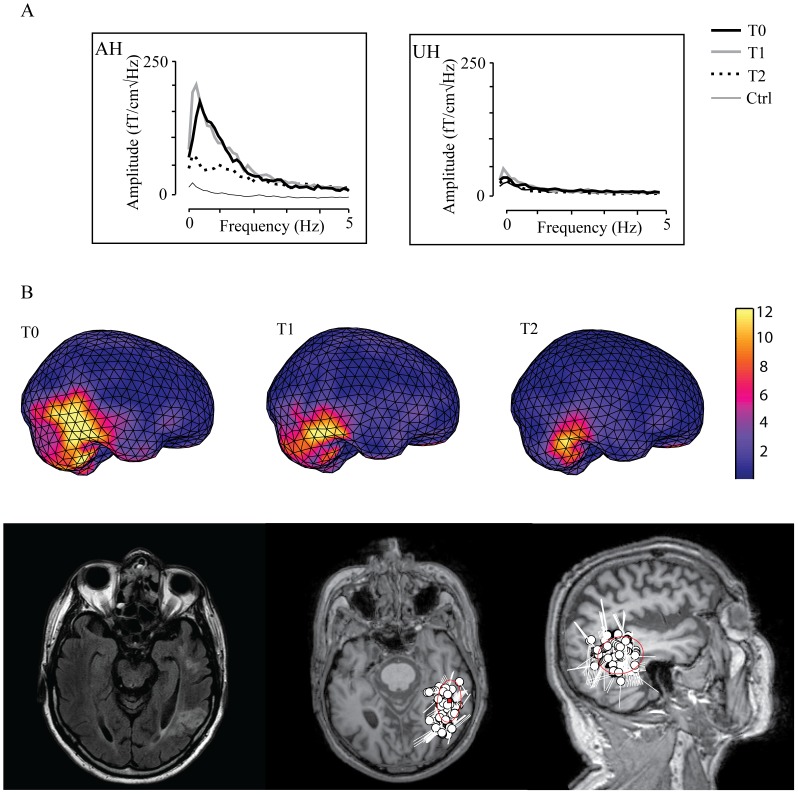
Spectrum and source localization of abnormal low-frequency magnetic activity (ALFMA). A) Amplitude spectra (eyes closed) of spontaneous 0–5-Hz brain oscillations in one patient and one control subject. Two channels from the amplitude spectra of spontaneous 0–5-Hz brain oscillations in one patient at T_0_, T_1_, and T_2_ and in one control subject. AH, channel from the perilesional area in the affected hemisphere. UH, channel from the corresponding region in the unaffected hemisphere. T_0_, 1 week; T_1_, 1month; T_2_, 3 months after stroke. B) Source localization of ALFMA in one patient *above* Source localization of ALFMA (arbitrary scale) projected on the surface of the standard brain volume (same patient as in A). *below* Source localizations of ALFMA projected on the MRI of the patient. Lesion localization is shown on the left.

Figure 5A illustrates the frequency spectra of spontaneous 0–5 Hz brain oscillations from two gradiometer channels overlying the affected (AH) and unaffected hemispheres (UH) in one patient at T_0_, T_1_, and T_2,_ and in one control subject. The spectra show a clear peak at ∼1 Hz in the AH in the patient at T_0_ and T_1_, and an elevated level of ALFMA still at T_2_. ALFMA were detected in seven out of 16 patients at T_0_, and in six patients at T_1_. In four patients ALFMA persisted still at T_2_. In six of the patients with ALFMA the lesion included the cortex, whereas one of the patients had a pure subcortical stroke.

Figure 5B illustrates the localization of the sources of ALFMA in one patient at T_0_, T_1_, and T_2_. Localization found with fdMCE corresponded well with the localization with ECDs. In patients with cortical lesions, the sources of ALFMA were localized in the perilesional cortex, whereas in the patient with a pure subcortical stroke the source was found in the cortical region overlying the lesion.

The lesion size was significantly larger in the patients showing ALFMA than in the rest of the patients (35±15 cm^3^ *vs*. 2±1 cm^3^, *p*<0.05). In addition, ALFMA seemed to be associated with worse clinical outcome. At T_0_, no statistically significant differences in the Peg test results or in the overall clinical outcome (measured with NIHSS) was detected between patients with or without ALFMA (101±13 *vs*. 75±16, *p* = 0.09 for Peg; and 5±1 *vs*. 3±1, *p* = 0.2 for NIHSS). However, the four patients with persistence of ALFMA had a worse clinical outcome at T_2_ than the rest of the patients (88±21 s *vs*. 33±3 s, *p*<0.001 for Peg; 3±1 *vs*. 1±0, *p*<0.05 for NIHSS).

In summary, ALFMA was detected in 7/16 patients at T_0_ and it persisted in four of these at T_2_. The patients with persistent ALFMA had a significantly worse clinical outcome at T_2_ than the rest of the patients.

## Discussion

### Temporo–parietal ∼10-Hz oscillations

In the patients, the strength of temporo–parietal ∼10-Hz oscillations was increased for the eyes-open condition in the AH at T_1_ and T_2_ as compared with the UH. In contrast, no such difference was observed for the eyes-closed condition. In the control subjects, no interhemispheric differences in the strength of temporo–parietal ∼10-Hz oscillations was detected neither for the eyes-open nor the eyes-closed condition, indicating that although the amplitude of spontaneous brain oscillations varies interindividually [2], the amplitude is rather similar in the two hemispheres in healthy subjects. Further, the interhemispheric ratio (AH/UH) of the strength of ∼10-Hz oscillations was significantly larger in the patients than in the control subjects, indicating that this interhemispheric difference, detected only in the patients, is a pathological phenomenon. The strongest sources of ∼10-Hz oscillations in the eyes-open condition were localized in the temporo–parietal region, clearly distinct from the occipital region, but slightly lateral to the typical rolandic area.

In addition to occipital and rolandic ∼10-Hz oscillations, spontaneous oscillations at 7–10-Hz have been detected in the temporal lobe (auditory tau-rhythm) [21] and in the parietal operculum, most likely in the secondary somatosensory cortex (SII; sigma rhythm) [22] in healthy subjects. The strongest sources of our prominent ∼10-Hz oscillations could agree with the location of the sigma rhythm. However, fdMCE analysis revealed that the amplitude of ∼10-Hz oscillations increased in bursts and had generators both in the rolandic region and in the parietal operculum. As the source localization and strength of ∼10-Hz oscillations varied strongly from time window to another used in spectral estimation, no exact separation between sources in the rolandic region and the parietal operculum could be made. Hence, we cannot rule out contributions of different temporo–parietal rhythms (such as the tau, sigma and rolandic ∼10-Hz rhythms) to the observed ∼10-Hz oscillations detected over the temporo–parietal region in our patients and control subjects, and thus we call this rhythm temporo–parietal ∼10-Hz rhythm. The occipital alpha rhythm is known to be strongly modulated by opening or closing the eyes [23], and some of its activity may be detected also at sensors above the temporo–parietal region. However, the interhemispheric difference in the strength of the temporo–parietal ∼10 Hz rhythm was detected only for the eyes-open condition, when the occipital alpha rhythm is dampened. Thus the increased temporo–parietal ∼10-Hz activity, described in the AH, likely represents a rhythm clearly distinct from occipital alpha.

Earlier studies on the strength of spontaneous ∼10-Hz oscillations after stroke have reported varying results: both decreases and increases of rolandic ∼10-Hz oscillations in the AH have been reported [4], [6], [8], [9]. In these studies ∼10-Hz oscillations were studied at the sensor level and no source modeling was performed; thus, non-rolandic contributions can not completely be ruled out.

No correlation between the strength of ∼10-Hz oscillations in the AH and clinical outcome has been found in earlier stroke studies, in line with the findings of the present study. Earlier studies have shown that post-stroke recovery mechanisms may differ between patients with cortical *vs*. subcortical strokes [24], [25]. In the present study, seven patients had a subcortical, three a pure cortical, and six a cortico–subcortical stroke. It may be that the lesion site affects the amplitude of ∼10-Hz oscillations, which could explain the lack of correlation between the strength of the ∼10-Hz oscillations and clinical recovery in the present study. Unfortunately, we were not able to demonstrate different effects of cortical *vs*. subcortical strokes as the separate groups were rather small. However, Pfurtscheller et al. [4] found enhanced ∼10-Hz oscillations in the AH in the acute state in patients with mild or moderate neurological deficits and months later in patients with slow clinical recovery from a severe neurological deficit. This enhancement was interpreted as a favorable sign for recovery. In our patients, the steepest clinical improvement was observed from T_0_ to T_1_. Accordingly, the ipsilesional enhancement of temporo–parietal ∼10-Hz was observed not earlier than at T_1_, suggesting that the enhanced rhythm may be linked with positive alterations in the somatosensory cortex. However, as no correlation between increased ∼10-Hz oscillations and clinical recovery was observed, this suggestion remains speculative and future studies are needed to evaluate the functional significance of increased ∼10-Hz oscillations during stroke recovery.

In healthy subjects, ∼10-Hz oscillations have been shown to be involved in the engagement and disengagement of specific brain regions in tactile discrimination tasks [26] and in somatosensory working memory performance [27]; ∼10-Hz activity has been shown to increase in task-irrelevant areas and to decrease in task-relevant areas. Thus ∼10-Hz activity is suggested to filter the inflow of sensory input with respect to its anticipated relevance [26]. The increase in ∼10-Hz activity has been linked to active inhibition of neuronal firing [28]. In the light of these findings, it may be that the enhancement of ∼10-Hz oscillations in the AH of our stroke patients could possibly be engaged in allocating resources for recovery mechanisms. However, this remains hypothetical, and needs to be elucidated in future studies.

Earlier studies have suggested slowing of both occipital alpha rhythm and rolandic ∼10-Hz rhythm in the affected hemisphere (AH) after thalamic [10] and middle cerebral artery territory strokes [6], [9], [10], [29]. In line, we observed deceleration of the occipital alpha rhythm in the AH of our stroke patients at T_2_. However, at group level, no such deceleration was observed in the temporo–parietal ∼10-Hz rhythm. This may be due to differences in sites of the lesions in the middle cerebral artery territory. Cortical rhythms are mainly generated in cortical areas [30], [31]. However, the thalamus has been suggested to play an essential role in driving the cortical rhythmic activity [32], [33]. Hence, disturbances in different sites of thalamocortical connections may affect the peak frequency of occipital alpha and temporo–parietal ∼10-Hz activity differently. The differences in the amplitude and frequency behavior of the occipital alpha and the temporo–parietal ∼10-Hz rhythm further emphasize the distinct generators and functional role of these rhythms.

### ALFMA

Earlier studies have indicated that brain lesions are often accompanied by abnormal electromagnetic activity at 1–4 Hz that can be directly measured with MEG [3], [13]. Animal studies have indicated that this abnormal activity is caused by partial cortical deafferentation [34], [35]. This theory was supported in a recent study, which combined MEG and diffusion tensor imaging (DTI) in patients with traumatic brain injury, and found a co-occurrence of axonal injury with ALFMA [11]. A combined MEG and proton magnetic resonance spectroscopic imaging study suggested an association between ALFMA and abnormal metabolic activity in preserved but dysfunctioning cortical neurons adjacent to the lesion [36]. However, the functional significance of these oscillations is still poorly understood.

Perilesional ALFMA has also been detected in stroke patients [12], [13], [37], but the relationship between ALFMA and the site or size of the lesion has remained unclear. Vieth et al. [13] detected ALFMA at 1–6-Hz range in patients with focal and superficial lesions. Similarly, Butz et al. [12] detected perilesional ALFMA at 1–3 Hz in 15 out of 23 patients in different stages after cortical strokes. In the present longitudinal study, ALFMA was detected in the acute phase in the affected hemisphere (AH) in 7/16 patients; 6/9 patients with a lesion extending to the cortex and only in 1/7 patients with pure subcortical stroke, in line with the earlier observations. These results suggest that cortical damage may predispose to the generation of ALFMA after stroke.

In adult rat thermal-ischemic lesions of the sensorimotor cortex induced ALFMA that was strongly correlated with axonal sprouting. Both ALFMA and axonal sprouting were blocked with tetrodotoxin infusion, suggesting that ALFMA may have a role in anatomical reorganization after a brain lesion [38]. In humans, no systematic relationship between the occurrence or persistence of ALFMA and clinical findings has been observed in earlier studies. In the present study, the patients with persistence of ALFMA had a worse clinical outcome at T_2_ than the rest of the patients. However, as the patient number with persistence of ALFMA was very small (4 patients), no definitive conclusions on the correlation between persistence of ALFMA and clinical outcome can be drawn. Whether ALFMA is a sign of severity of neuronal damage or a signal of still ongoing plastic reorganization needs to be elucidated in future studies.

### Predictive value of spontaneous brain oscillations

The strength of spontaneous gamma oscillations (>34 Hz) in the AH and the strength of delta (2.0–3.5 Hz) oscillations in the UH in the acute phase after stroke have been suggested to predict recovery [5]. In the present study, no clear spectral peaks in the gamma range were detected in the patients or in the control subjects, neither did we detect delta peaks in the UH of the patients. As the MEG/EEG signal strength typically decreases with increasing frequency, it may be difficult to separate gamma band oscillations from the background and sensor noise and possible muscular artifacts, due to the low signal-to-noise ratio. Most reports of gamma oscillations have been associated with event-related phenomena such as visual processing or motor tasks [39], [40] that synchronize the envelope of gamma oscillations and thus increase their detectability. Resting-state gamma oscillations are more difficult to separate from background and sensor noise and were probably therefore not observed in our patients. In contrast, ∼10-Hz activity is a very robust signal, which is detected practically in every subject. In 13/16 of our well-recovering patients, enhancement of AH ∼10-Hz activity was detected at some time point. Thus increased ∼10-Hz activity may have a functional role in stroke recovery. Future studies should aim at investigating if this enhancement could be used to predict the outcome from stroke.

In conclusion, our results show that stroke causes perilesional ALFMA, which persists in some patients and which may be associated with the severity of the stroke and poor recovery. Moreover, the temporo–parietal ∼10-Hz oscillations were enhanced in the affected hemisphere during recovery of our patients, which may be a favorable sign for recovery. Future studies will show, whether temporo–parietal ∼10-Hz oscillations can be used to predict the outcome from stroke
